# Control of Blood Coagulation by Hemocompatible Material Surfaces—A Review

**DOI:** 10.3390/bioengineering8120215

**Published:** 2021-12-15

**Authors:** Janna Kuchinka, Christian Willems, Dmitry V. Telyshev, Thomas Groth

**Affiliations:** 1 Department Biomedical Materials, Institute of Pharmacy, Martin Luther University Halle-Wittenberg, 06120 Halle (Saale), Germany; janna.kuchinka@pharmazie.uni-halle.de (J.K.); christian.willems@pharmazie.uni-halle.de (C.W.); 2 Institute of Biomedical Systems, National Research University of Electronic Technology, Zelenograd, 124498 Moscow, Russia; telyshev_d_v@staff.sechenov.ru; 3 Laboratory of Biomedical Nanotechnologies, Institute of Bionic Technologies and Engineering, I.M. Sechenov First Moscow State University, 119991 Moscow, Russia; 4 Interdisciplinary Center of Materials Science, Martin Luther University Halle-Wittenberg, 06120 Halle (Saale), Germany

**Keywords:** coagulation, blood platelets, complement system, blood-material interaction, biomedical devices, surface modification, hemocompatibility

## Abstract

Hemocompatibility of biomaterials in contact with the blood of patients is a prerequisite for the short- and long-term applications of medical devices such as cardiovascular stents, artificial heart valves, ventricular assist devices, catheters, blood linings and extracorporeal devices such as artificial kidneys (hemodialysis), extracorporeal membrane oxygenation (ECMO) and cardiopulmonary bypass. Although lower blood compatibility of materials and devices can be handled with systemic anticoagulation, its side effects, such as an increased bleeding risk, make materials that have a better hemocompatibility highly desirable, particularly in long-term applications. This review provides a short overview on the basic mechanisms of blood coagulation including plasmatic coagulation and blood platelets, as well as the activation of the complement system. Furthermore, a survey on concepts for tailoring the blood response of biomaterials to improve the hemocompatibility of medical devices is given which covers different approaches that either inhibit interaction of material surfaces with blood components completely or control the response of the coagulation system, blood platelets and leukocytes.

## 1. Introduction

Blood-contacting medical devices, such as catheters, blood linings, vascular grafts, stents, heart valves, and artificial organs are widely used in clinical medicine to treat different diseases. Several materials, with a variety of properties, are used for these devices, including metals, polymers, ceramics, and composites [1]. For example, titanium alloys are frequently used biomaterials because of their excellent biocompatibility, corrosion resistance (due to a passivating natural oxide layer), inertness, and low cost [2,3]. They also find application as part of ventricular assist devices [4]. Other metal alloys such as nitinol, cobalt chromium alloys or stainless steel are used for vascular stents and as components of artificial heart valves [5]. Polymers are widely applied in medical devices due to the adaptability of their mechanical and chemical properties to different applications. For example, polyurethanes (PU) are characterized by their excellent mechanical properties and good blood compatibility [6,7,8]. PUs have been used as components of artificial hearts, while polymers such as polytetrafluoroethylene (PTFE) and polyethylene terephthalate (PET) are used as artificial, large-diameter blood vessels due to their outstanding mechanical properties, long-term stability and relatively good blood compatibility [5]. Silicone rubber, polyolefines like polypropylene and polyethylene, PUs and other polymers are used in different types of blood linings and catheters [5]. Cellulose, polysulfones, polypropylene and other polymers are used for making membranes for hemodialysis and oxygenators in artificial organs such as artificial kidneys, artificial lungs and cardiopulmonary bypasses [9,10]. Ceramic materials can be found primarily as part of artificial heart valves and have limited application potential in this area. More detailed information on the wide range of biomaterials for blood-contacting medical devices can be found elsewhere [1].

However, none of these material classes listed above are sufficiently compatible with blood [6]. Therefore, systemic anticoagulation (e.g., by use of heparin) is required during the application of blood-contacting devices such as artificial heart valves, in hemodialysis and others. Particularly in long-term application of devices the use of anticoagulants is critical because of the increased risk of bleeding [11,12], which requires better blood-compatible biomaterials that do not require long-term anticoagulation. Consequently, there is a need for strategies to improve the hemocompatibility of bulk materials by, e.g., specific synthesis, blending of materials or by modifying the biomaterial surface to control the blood response [13,14,15,16]. Hence, understanding the interaction of blood components with foreign biomaterials and the effect of material surface properties represent important prerequisites for the development of blood-compatible biomaterials [17]. Various approaches exist to make hemocompatible surfaces, ranging from the passivation of surfaces over functionalization with bioactive molecules to endothelialization.

This review will provide a brief overview on protein adsorption as first event, followed by a discussion of the mechanisms of coagulation and platelet activation, and a short description of complement activation. Furthermore, we will provide here a survey on different modification strategies for biomaterials to improve the hemocompatibility of medical devices.

## 2. Protein Adsorption

Thrombus formation on blood-contacting medical devices is a complex process. Typically, the contact of foreign biomaterials with blood immediately leads to the adsorption of plasma proteins on the biomaterial surface [17,18]. This process takes place through physical interaction forces like Coulomb and van der Waals forces, hydrogen bonding, and hydrophobic interactions, leading to the reversible or irreversible adsorption of plasma proteins [19]. It will be discussed later in more detail that this can be followed by the activation of coagulation including the clotting system and blood platelets. In addition, activation of innate immunity such as the complement system and monocytes can also have a promoting effect on coagulation and inflammatory response. It should be noted that the adsorption process and the dominating interactions are dependent both on the surface properties of the foreign material, as will be lined out below in some more detail, but also on the health state of the patients. For example, patients with coronary heart diseases or those with inherited or acquired problems of their coagulation system may have an increased incidence to develop thrombotic complications [20,21].

Protein adsorption on foreign surfaces starts with the adsorption of smaller and more abundant proteins such as human serum albumin which is driven by Fickian diffusion [22]. For example, albumin can be mostly found in such adsorption layers due to its lower molecular weight (LMW) of 66 kDa and high plasma concentration between 3.5–5 g/dL [23]. However, such LMW proteins can be displaced partly by proteins of higher molecular weight (HMW), even if they are less abundant, because of their higher affinity to the surface. Leo Vroman discovered that there is a sequence of protein adsorption from LMW to HMW proteins such as albumin displaced by immunoglobulins, displaced by fibrinogen and then high molecular weight kininogen (HMWK) [24]. This change in protein layer composition, known as the Vroman effect, is restricted to more hydrophilic surfaces [25,26]. More hydrophobic surfaces, which represent most of the common synthetic polymers, lead to a rather irreversible adsorption of proteins. Overall, plasma protein adsorption is dependent on the character of the surfaces, such as their wetting properties and charge density [27]. In this regard, it is interesting to note that many plasma proteins have hydrophilic domains on their surface and a hydrophobic core [18]. However, epitopes with hydrophobic amino acids can also be located at the outer part of proteins driving adsorption on hydrophobic surfaces [27]. Adsorption of proteins is mostly followed by their conformational change with time which usually results in the unfolding of proteins, affecting their biological functions [19,28]. This is also dependent on protein size. Norde and Lyklema separate “hard” (smaller, more stable) from “soft” (larger, less structure-stable) proteins, which means that particularly larger proteins have a higher tendency to adsorb and unfold [27]. The final adsorption layer contains a multitude of proteins including fibrinogen and other coagulation factors, immunoglobulins, as well as adhesive proteins like fibronectin and vitronectin [29,30]. Because of the large number of different proteins present in human plasma (more than 1000) [31], a prediction of protein adsorption regarding composition and the functional state of the adsorption layer is impossible unless one is able to achieve selective adsorption of specific proteins such as albumin [32] or block their adsorption completely [33]. The presence of the adsorption layer can be related to the activation of the blood coagulation and complement system but also to adhesion and activation of blood platelets and leukocytes which represent all initiators of undesired blood–material interactions, such as thrombosis and inflammation [34,35]. The mechanism of thrombus formation and complement activation will be described in subsequent sections of this review.

It is important to understand that protein adsorption and activation of blood components are strongly dependent on the nature of the biomaterial surface. Thus, chemical and physical properties, including the molecular composition, surface wettability, charge and topography of the surface are important factors to consider.

While hydrophobic surfaces are characterized by an absence of polar or charged groups, hydrophilic surfaces are based on their presence which makes them attractive for water molecules and other polar or charged species (e.g., ions, amino acids, lipids) [36]. The terms hydrophobic and hydrophilic are defined by water contact angles larger or smaller than 90° [37]. Typically, hydrophobic biomaterials like polytetrafluorethylene (PTFE), polyethylene terephthalate (PET), polyethylene, polypropylene and other polymers are associated with stronger protein adsorption [5] caused by the interaction of the surface with hydrophobic domains of the proteins. This is related to entropy-driven displacement of clusters of water molecules from hydrophobic surfaces and protein domains, also called the “hydrophobic effect” [38]. On the other hand, polar, hydrophilic surfaces in general show a lower adsorption of proteins due to repulsive forces caused by the presence of a thin, more or less strongly-bound water layer on the material surface [5]. If this water layer is stabilized through polar interactions, e.g., hydrogen bonds, it leads to the so-called repulsive hydration force [39], which means protein adsorption is thermodynamically suppressed [40]. Specific examples of such surfaces are the membrane of red blood cells which is rich in phospholipids with polar or zwitterionic head groups particularly phosphatidylcholine [41,42], and surfaces that possess many hydroxyl groups [43,44]. Another repulsive force is represented by the so called “steric repulsion” that is related to the energetically unfavored compression of hydrophilic macromolecules immobilized on surfaces [45]. Again, the glycocalyx of cells can serve as an example provided by nature of control of the adsorption of proteins and cells, one which has also been exploited to control protein adsorption on man-made materials [46].

The type of charge also influences the protein adsorption on hydrophilic surfaces. Negatively-charged surfaces typically lead to a lower extent of protein adsorption than positively-charged surfaces because many proteins have a net negative charge at physiological pH value, which provides a repulsive force; though the rather high salt concentration in body fluids decreases the range of the repulsive Coulomb force [27]. Positively-charged material surfaces greatly increase protein adsorption [47] and may also provoke conformational changes of proteins. Because of the presence of protein domains that may still be oppositely charged, even though the net charge may create an overall repulsive Coulomb force against the surface, protein adsorption can still happen due to attractive forces between the oppositely charged protein domains and the surface [27].

Protein adsorption is also influenced by surface topography, particularly when it is at the nanoscale [48]. Texture at the microscale and nanoscale increases the surface area which increases the quantity of adsorbed proteins in general. It has been observed that smooth or polished surfaces show lesser interactions with proteins, whereas roughness leads to an increased protein adsorption [49,50,51,52,53]. In this regard, nanometric surface features also affect protein adsorption and cell adhesion [54,55]. Moreover, micrometer-sized air bubbles captured on rough surfaces provide an interface of high energy difference to the fluid phase of blood that can attract proteins and also change their conformation [56].

Therefore, more blood-compatible materials are characterized by surfaces of low interfacial energy (hydrophilic) that may also repel proteins by steric repulsion and are rather smooth to avoid capture of air bubbles and so reduce adsorption of plasma proteins. However, this is difficult to achieve with most standard biomaterials due to their required bulk physical properties, such as mechanical properties, glass transition temperature, transparency, conductivity, permeability, ability of sterilization, but also because of the low costs required, particularly in large scale application (e.g., tubing, catheters, dialyzers, etc.).

In the following section, we will provide a short overview on the mechanisms of coagulation, thrombus formation, and fibrinolysis with some discussion of effects exerted by foreign surfaces. Moreover, activation of the complement system will be briefly discussed.

## 3. Coagulation, Platelet and Complement Activation on Foreign Materials

**Figure 1:**
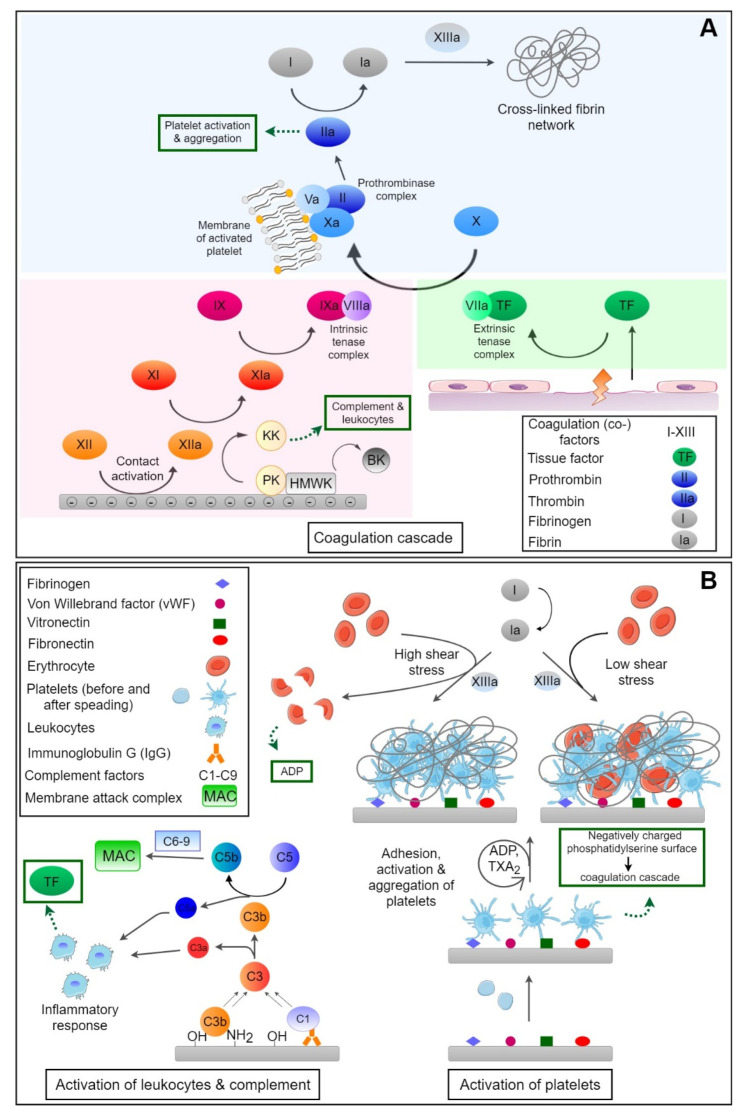
(**A**) Activation of coagulation by the intrinsic system through autoactivation of factor XII (FXIIa) with amplification by kallikrein (KK) and the extrinsic system after injuries through tissue factor (TF) leading both to activation of factor X (FXa). This is a component of a prothrombinase complex formed on the cell membrane of activated platelets exposing phosphatidylserine. KK also splits high-molecular-weight kininogen leading to release of vasoactive bradykinin (BK). Thrombin (FIIa) causes activation of fibrinogen leading to fibrin polymerization which is cross-linked by factor XIIIa. Thrombin is also the strongest agonist of platelets leading to their activation and aggregation. (**B**) Activation of complement system by covalent reaction of complement factor C3b with nucleophilic groups on material surfaces (e.g., OH), bound antibodies, and immune complexes through factor C1q with generation of C3 and C5 convertase and release of anaphylotoxins C3a and C5a with activation of leukocytes, as well as the release of TF connecting complement with coagulation. As the final step of complement activation the membrane attack complex (MAC) is formed. Adhesion and activation of platelets can happen through adsorbed plasma proteins followed by shape change, release of platelet agonists like thromboxane A2, and ADP. Platelets provide a procoagulant surface for the formation of the prothrombin complex connecting them to the coagulation cascade. Moreover, thrombosis is dependent on flow conditions with formation of red thrombi and inclusion of red blood cells into the fibrin network at low shear stress, while high shear stress leads to the formation of white thrombi and can also induce rupture of red blood cells that release ADP-activating blood platelets.

The healthy endothelium actively resists thrombosis. Under physiological conditions, the endothelium regulates thrombus formation and dissolution through the expression and secretion of several antithrombotic and fibrinolytic factors. These include thrombomodulin, prostaglandins, heparan sulfate and nitric oxide to prevent thrombosis as well as tissue plasminogen activator (tPA), urokinase plasminogen activator (uPA) and plasminogen activator inhibitors to regulate fibrinolysis [57]. In contrast to the healthy endothelium, foreign surfaces may cause thrombus formation and inflammation, mainly triggered by initially adsorbed proteins but also depending on the flow conditions if they are non-physiological with too low or too high shear forces [58,59]. Thrombus formation on foreign materials involves several steps, such as the adsorption of proteins (among them proteins from the coagulation cascade) followed by autoactivation of factor XII (FXII) and adhesion, activation and aggregation of blood platelets, as well as activation of the complement system. Figure 1 provides a survey on these processes.

### 3.1. The Coagulation Cascade

The blood coagulation cascade consists of a number of proteases which activate each other, leading to the formation of thrombin and, subsequently, fibrin polymerization [23,60]. A simplified scheme of coagulation is shown in Figure 1A. It can be divided in two major pathways, the extrinsic and the intrinsic pathway. These two systems are activated by different mechanisms and merge with the activation of factor X (FX) in the common pathway, where a fibrin clot, the end product of the coagulation cascade, is generated [61].

The intrinsic pathway, also known as contact activation of the coagulation cascade, is the more critical pathway with respect to blood-contacting medical devices [62,63]. It is initiated upon adsorption of FXII (Hageman factor [64]) and HMWK on negatively-charged surfaces, whereby FXII undergoes a conformational change and subsequent autoactivation to FXIIa. Here ‘a’ stands always for the activated clotting factors. It is known that particularly negatively-charged surfaces and molecules can trigger autoactivation of the Hageman factor [65], which can happen on negatively-charged collagen fibrils of the sub-endothelium which are exposed after injuries [66] but particularly by exposure of negatively-charged phospholipids of damaged endothelial cells, activated blood platelets and neutrophils, and released polyphosphates and extracellular DNA [67,68]. FXIIa activates factor XI (FXI) to FXIa which then converts factor IX (FIX) to FIXa. Cofactor VIIIa (FVIIIa) and FIXa form a complex (FIXa:FVIIIa, intrinsic tenase complex), which in turn activates FX to FXa. Furthermore, FXIIa converts prekallikrein (PK) to kallikrein (KK) which activates more FXII, promoting its own feedback activation [69]. There is a multitude of examples from in vitro studies and clinical applications of biomaterials showing activation of the contact system of plasmatic coagulation by material surfaces. For example, it has been shown in vitro that negatively charged groups of different types of copolymers of polyacrylonitrile membranes promote activation of the contact system [16]. This was strongest when acrylic acid was used as a comonomer but also when hydroxyethylacrylate was used during polymerization. These effects were related to a high content of anionic carboxylic groups and more negative zeta potentials, something which supports the common finding of contact activation by negatively charged surfaces. As the presence of negative charges prevents protein adsorption, as mentioned above, but can also cause an increase in contact system activation if the charge density is too high, it is imperative that the charge density on a material surface is carefully tuned. For example, iron oxide particles, potentially developed as magnetic resonance imaging (MRI) contrast agents, can provoke a strong activation of the contact system unless they are chemically modified with amino groups that increase their zeta potential [70]. During contact activation, KK degrades HMWK, which leads to the release of the vasoactive peptide bradykinin (BK) that normally has a short lifetime and is involved in the regulation of blood pressure [71]. In this regard, it has been observed that polymer membranes made of a copolymer of acrylonitrile with allylsulfonate as comonomer (AN69S) caused serious complications in patients under medication with angiotensin converting enzyme (ACE) inhibitors [72]. The use of allysulfonate introduces anionic charges to the membrane material which can trigger contact activation [73]. ACE inhibitors that are prescribed to decrease blood pressure in patients suffering from chronic kidney failure [74] block degradation of BK [75]. It was observed that patients exposed to large surface areas of AN 69S dialyzers developed a shock syndrome due to extreme hypotension with sometimes fatal outcomes [72]. Hence, the presence of strong negative surface charges on biomaterials can be considered a potential trigger of contact activation and should be avoided, particularly when large surface areas (hemodialysis, ECMO) encounter the blood of patients.

The extrinsic pathway (see Figure 1A) is activated by vascular injury, when cells bearing the tissue factor (TF) are exposed to blood [76]. TF is located in the ECM underneath the endothelial cells [77], and in fibroblasts, and smooth muscle cells [78]. TF forms a complex with activated coagulation factor VII (FVII). This complex (TF:FVIIa, extrinsic tenase complex) subsequently activates FX to FXa. However, the TF:FVIIa complex also activates FIX to FIXa which is part of the intrinsic coagulation pathway [79,80]. For a long time it was assumed that the extrinsic pathway plays no relevant role in blood–material interaction but it seems that blood contact with an artificial material can be a potential activator of the extrinsic pathway due to TF expression by monocytes [81].

The extrinsic and intrinsic pathway both activate FX to FXa which constitutes the first step of the common pathway. FXa in complex with activated cofactor V (FVa) converts prothrombin to thrombin which then polymerizes fibrinogen into fibrin [69]. It has been found that thrombin could directly activate FXI which in turn leads to more thrombin generation [82]. Factor XIIIa (FXIIIa) is cross-linking fibrin which forms a more stable fibrin network and is the primary structure of blood clots. The cross-linked fibrin fibers entrap blood cells, e.g., platelets or red blood cells, which leads to the formation of red or white thrombi depending on the flow conditions with low or high shear force [83].

The activation of the coagulation system is regulated by counterplayers such as antithrombin III (AT III) which inactivates thrombin and FXa in particular by forming a complex where the active site of the clotting enzyme is blocked but also inactivates other activated serine proteases (e.g., kallikrein) [84,85]. The complex formation of thrombin and FXa with AT III is relatively slow, but it is significantly accelerated in the presence of heparan sulfate which occurs naturally on endothelial cells or when heparin is present. AT III affinity to thrombin is amplified by a factor of about 1000 by heparin which is exploited in its clinical application [86]. It is also of note that non-fractionated heparin supports formation of a ternary complex of ATIII, with activated serin proteases such as thrombin, with the subsequent release of the ATIII-clotting enzyme complex. Another physiological inhibitor of coagulation is the tissue factor pathway inhibitor (TFPI) which is able to inactivate FXa and the TF:VIIa complex [81]. TFPI is present in plasma on the vascular endothelium, on platelets, and also may be released by activated monocytes [87]. Thrombomodulin is a protein that is localized on endothelial cells and is also involved in the inhibition of coagulation but also possesses an anti-inflammatory activity. Hence, recombinant thrombomodulin has been recently introduced into the clinical practice as an anticoagulant and anti-inflammatory drug for the treatment of patients with sepsis-induced coagulopathy [88]. It forms a complex with thrombin which activates protein C, a vitamin K-dependent protein. Activated protein C in turn forms a complex with cofactor protein S, inactivating FVa and FVIIIa. Ref. [89] Hence, vitamin K antagonists have been used for decades to prevent thromboembolism in patients [90].

### 3.2. Fibrinolysis

Fibrinolysis describes the process of clot destruction by plasmin as a primary fibrinolysin which degrades insoluble fibrin clots. Like the coagulation cascade, fibrinolysis is tightly regulated under physiological conditions. The activation involves the conversion of the inactive precursor protein plasminogen into the active serine protease plasmin triggered by two endogenous activators; the proteases tissue plasminogen activator (tPA) and urokinase plasminogen activator (uPA) [91]. tPA is produced and released by endothelial cells, while uPA is the product of monocytes, macrophages, and urinary epithelium [91]. Both have a very short half-life of several minutes due to the presence of inhibitors such as plasminogen activator inhibitor-1 (PA1) or thrombin-activable fibrinolysis inhibitor (TAFI) [92]. Fibrin fibers represent a key activation site for the fibrinolysis through the binding of tPA and upregulation of its catalytic activity to activate plasminogen up to a factor of 500 in comparison with tPA in plasma. In contrast to rapid tPA, uPA and plasmin neutralization in plasma by circulating inhibitors, the surface of endothelial cells and the fibrin clot represent a safe site for fibrinolysis. In particular, cell surface receptors such as the uPA receptor and Annexin A2 complex bind plasminogen and activators on the surface of endothelial, monocytes and other cells [91]. Changes in fibrinolysis are related to insufficient hemocompatibility of membrane materials used in hemodialysis and cardiopulmonal bypasses when a large surface area encounters the blood of patients [93,94]. Hence, beside the activation of the clotting system through the contact of blood with foreign surfaces, excessive activation of fibrinolysis may occur which increases the risk of bleeding in patients.

### 3.3. Platelet Adhesion, and Activation

The adhesion and activation of platelets in the human body occurs after damage of the healthy endothelium which displays the sub-endothelial basal lamina with collagens, but also by exposure of atherosclerotic plaques, and the release of platelet agonists like thrombin, adenosine diphosphate (ADP), thromboxane A_2_ (TXA_2_) and others [57]. This process is called primary hemostasis because platelets form a plug that helps to stop bleeding followed by secondary hemostasis with activation of the coagulation system (see Figure 1B) [95].

Platelets are small spherical to discoid shaped anuclear cells, ordinarily circulating in blood. They are characterized by the presence of different types of granula in their cytoplasm among them those that release their content upon activation. Here, we have α granula that contain several coagulation factors, but also plasminogen, von Willebrandt factor (vWF), and fibronectin (FN) which facilitate platelet adhesion and spreading. Dense granules contain ADP, calcium, and serotonin that play an important role for platelet activation, coagulation, and the regulation of blood pressure, respectively. The distribution of membrane lipids is asymmetric with phosphatidylserine (PS) inside the cell membrane which can be translocated to the outer membrane surface during platelet activation [96]. There it serves as cofactor in the formation of the prothrombin complex which is one of the contributions of platelets to secondary hemostasis (coagulation) [97,98]. Furthermore, the release of prostaglandins like TXA_2_, but also ADP, and other agonists by activated platelets amplifies their adhesion, activation, and aggregation. The process is related to signal transduction events via G-coupled receptors that transmit signals for the reorganization of the cell cytoskeleton during platelet shape change and aggregation [99], as well as the activation of glycoprotein IIb/IIIa, permitting the binding of fibrinogen from plasma as linker during platelet aggregation; something which is also related to a contraction of the platelet plug as a mechanism to reduce or stop bleeding [60]. The formed platelet aggregates are stabilized by fibrin, forming a platelet-fibrin thrombus. It is also noteworthy that platelet activation and local aggregation is promoted by thrombin which represents the strongest platelet agonist [100,101].

On foreign surfaces, on the other hand, platelet activation mainly takes place through platelet adhesion due to adsorbed plasma proteins. Foreign surface-induced platelet adhesion and activation is initiated by surface-adsorbed proteins, such as von Willebrand factor (vWf), and fibrinogen, but also fibronectin, and vitronectin [102,103,104]. A simplified scheme of platelet interactions in contact with biomaterials and subsequent thrombus formation is depicted in Figure 1B. Blood platelets can bind to specific amino acid sequences, e.g., RGD (Arg-Gly-Asp) of adsorbed proteins via cell adhesion receptors on the platelet surface [102,105]. These include, i.e., the glycoprotein (GP) Ib/IX receptor, mediating platelet adhesion by binding vWf, and integrin α_IIb_ß_3_ (GPIIb/IIIa) receptor which support platelet adhesion and aggregation by binding fibrinogen [99,106]. Particularly adsorbed fibrinogen represents an activator of blood platelets related to its concentration and conformational changes [13,107]. For example, it has been shown that adhesion, spreading, and aggregation of platelets is increased on hydrophobic polymer membranes (e.g., polysulfone) but suppressed on hydrophilic membranes made of cellulose (Cuprophane) which was related to the quantity and conformation of adsorbed fibrinogen [108].

### 3.4. Complement Activation

The protein layer adsorbed on foreign surfaces not only influences platelet adhesion and activation of the coagulation system. It may also induce the activation of the complement system and leukocytes which supports also local and systemic inflammatory responses [109]. The complement system consists of several proteins which activate each other in a cascade-like process, like the coagulation cascade. Complement activation can occur through three different pathways: the classical, alternative, and lectin pathway, of which the alternative pathway is the most relevant in relation to blood-contacting biomaterials. Adsorption of complement factors but also other proteins, in particular immune globulins, represent a trigger of complement activation [110].

The alternative pathway (see Figure 1B) can be directly initiated by foreign surfaces through spontaneous hydrolysis of the complement factor C3 which can be followed by a covalent reaction with the surface [111,112,113]. The major fragment C3b can covalently bind to surfaces with hydroxyl or amino groups [114] which initiates the complement activation of the alternative pathway with formation of C5 convertase, finally leading to the formation of a membrane attack complex (MAC) which forms a pore in the wall of bacteria leading to their destruction [115]. Indeed, during activation of the complement system by blood-contacting biomaterials this can also lead to lysis of red blood cells [116] which then release ADP as a strong activator of blood platelets and hemoglobin. However, activation of the classical pathway of the complement system is also triggered by the binding of complement factor C1q to immune complexes or adsorbed immune globulins [110]. Hence adsorption of immune globulins on material surfaces also represents a potential trigger of complement activation. As a result of complement activation, a release of anaphylotoxins C3a and C5a can occur with systemic responses like shock syndrome but also local adhesion and activation of leukocytes may be promoted on the material surface with adverse local (inflammation) and also systemic effects (e.g., fever) [58]. It has also been observed that the activation of leukocytes is closely correlated with the release of soluble C5a while their adhesion is related to adsorption and activation of complement factor C3 [114]. Previous studies have also shown that hemodialysis membranes made of cellulose activate the alternative pathway of the complement system due to the presence of hydroxyl groups [117], something related to leukopenia and fever. Masking of hydroxyl groups by other functionalities like acetates reduced the complement activation greatly and made the membranes more blood compatible [118].

A further effect of complement activation is the amplification of coagulation and inhibition of fibrinolysis mainly through factor C5a which induces the expression of TF and plasminogen activator inhibitor I (PAI-I) [110,119]. On the other hand, the coagulation system can increase complement activation through FXIIa that acts by C1 cleavage while thrombin can activate C5 directly leading to release of C5a. Therefore, material surfaces should also be designed to avoid the activation of the complement system, particularly if the intended application requires a large surface area interacting with the blood of patients as is the case in hemodialysis or ECMO.

## 4. Design of Blood-Compatible Surfaces

**Figure 2:**
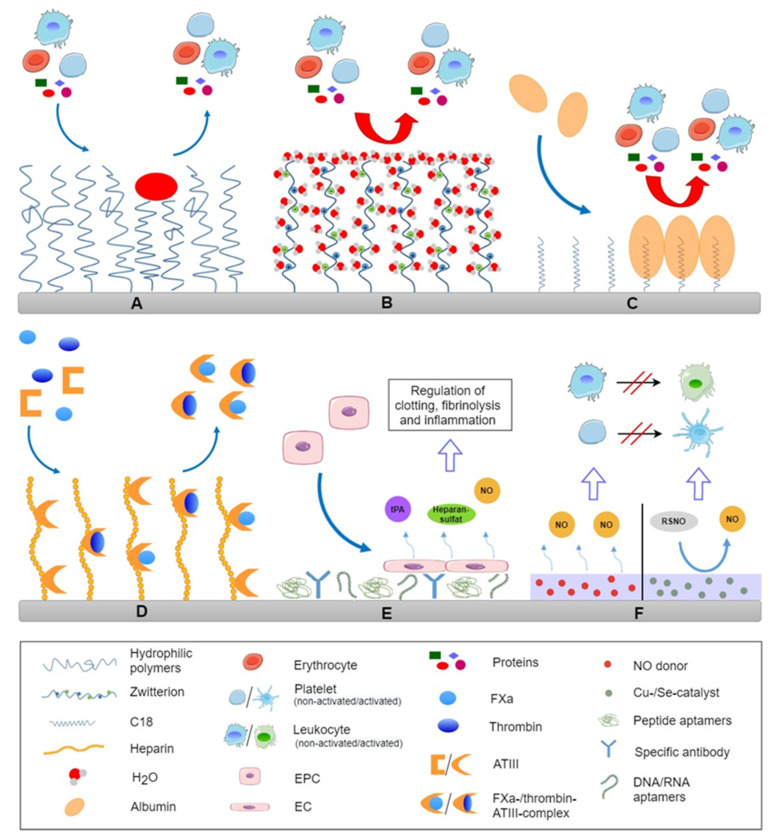
Survey on different design of blood compatible surfaces by steric repulsion through immobilization of mobile hydrophilic macromolecules (**A**), zwitterionic molecules that lead to tight binding of water molecules causing strong repulsive hydration forces (**B**), preferential binding of a passivating protein layer from plasma, like immobilization of C18 fatty acids for preferential adsorption of albumin (**C**), end-on immobilization of heparin for binding anti-thrombin III for inactivation of thrombin (FIIa), factor Xa and other coagulation factors (**D**), immobilization of antibodies or aptamers for preferential adhesion of endothelial progenitor cells from circulation for formation of an endothelial lining (**E**) and NO releasing/generating surfaces for control of leukocyte and platelet activation (**F**).

To guarantee a safe use of blood-contacting medical devices, surface induced adverse events such as undesirable blood coagulation, thrombus formation, and complement activation must be avoided. Surface modification through coatings or by physical or chemical functionalization of the surface of biomaterials plays a key role in preventing these complications. As outlined before, device-related thrombus formation is triggered through protein adsorption as first step of activation of the coagulation system and blood cells (e.g., platelets and leukocytes). In particular, the adsorption of coagulation factors, such as FXII, HMKW and fibrinogen, but also complement factors, immune globulines and adhesive proteins (vWF, FN, VN), must be suppressed or controlled to avoid the activation of the different defense systems and blood cells. Thus, methods to obtain hemocompatible surfaces have focused on modifications to resist adsorption of blood proteins and adhesion of cells, as well as on the active inhibition of the coagulation cascade and platelet activation. In general, one can differentiate between passive and active surfaces. Passive or bioinert surfaces act as a barrier between the foreign biomaterial and the blood, whereas bioactive surfaces directly interact with blood components. A survey on the different concepts of blood compatible surfaces is shown in Figure 2, while examples of different commercial devices based on these concepts are shown in Table 1.

### 4.1. Passivating Surfaces

Modification strategies to make biomaterial surfaces resistant against non-specific protein adsorption, and blood cell adhesion are based on changes in surface properties to reduce the interfacial energy that promotes adsorption of proteins and create repulsive barriers against the attachment of proteins and cells which is illustrated in Figure 2A,B. This can be achieved by repulsive surface charges, making super-hydrophilic surfaces, as well as layers of hydrophilic macromolecules to exploit hydration forces and steric repulsion [62]. These kinds of modifications are related to chemical treatment of material surfaces, grafting of molecules or coatings. Both inorganic and organic surface modifications find application in the improvement of blood compatibility of medical devices.

#### 4.1.1. Inorganic Coatings

Inorganic modifications, like metal oxides, metal nitrides, and carbon-based coatings are well established in the field of blood contacting biomaterials, and specifically metals, find application in medical devices like blood pumps, artificial heart valves and stents [28].

Titanium and its alloys are widely used metals for implanted medical devices due to their excellent biocompatibility. Their biocompatibility is based on a natural oxide layer which is formed when the titanium surface is exposed to air [120]. The good blood compatibility is related to the surface energy and semiconductor properties, and depends on the thickness of the titanium oxide film, with thicker films being more hemocompatible [5]. It has also been claimed that the n-type semiconductor properties of titanium oxide surfaces are connected to low platelet adhesion and activation [121]. For example, Nan et al. demonstrated that artificially fabricated titanium oxide films, synthesized by ion beam enhanced deposition, improved the hemocompatibility of titanium [122]. Titanium nitride (TiN) coatings have also been used for a long time in medical devices like heart valves, heart assist devices and heart pumps [6]. TiN shows an improved hardness of the surface and good blood-compatible behavior, as does TiO. The coatings can be made by, e.g., physical or chemical vapor deposition, or energetic nitriding, and film deposition techniques [123,124,125]. TiN surfaces prevent thrombus formation to a high extent in a similar manner as TiO coatings but show a lower performance regarding wear resistance [126,127].

A widely established coating to improve the hemocompatibility of medical devices such as artificial heart valves, vascular stents, and ventricular assist devices [128] is the diamond-like carbon (DLC) coating. It can be used for a wide range of materials, e.g., titanium, titanium alloys, and polymeric materials receiving much attention due to several advantages [129,130,131]. This kind of coating shows a high strength and smoothness, chemical inertness, minimal wear, a low frictional coefficient, excellent biocompatibility, and hemocompatibility [5]. There are multiple methods for the preparation of DLC surfaces including chemical vapor deposition, pulsed laser deposition, direct ion beam deposition, cathodic arc deposition, magnetron sputtering, and plasma source ion deposition [132]. In several studies it has been demonstrated that biomaterials modified with DLC, showed a reduction of platelet adhesion on surfaces [129,133]. The good hemocompatibility of the DLC coating was attributed to its smooth surface, but particularly to its sp2/sp3 ratio leading to lesser protein adsorption, and a decrease of platelet adhesion and spreading [133,134]. However, DLC surface coatings also have some limitations including the risk of micro-cracks which can be formed on the surface [6]. These cracks promote thrombus formation and can be avoided using composites to incorporate elastic features. Moreover, there are stability problems through carbide formation in the presence of iron which is present in blood.

An alternative carbon-based coating with similar mechanical and biological properties like DLC is the boron-carbon-nitrogen coating [135]. Another carbon-based modification is pyrolytic carbon film. Manufactured by chemical vapor deposition, these coatings are used for medical devices like stents, vascular grafts, and artificial heart valves [58]. However, it should be mentioned that although in vitro and animal studies showed low platelet adhesion, clinical studies did not show the desired effect in long-term applications [136,137].

#### 4.1.2. Organic Coatings

Surface modification with organic molecules to control the interaction between proteins and cells with surfaces include the incorporation of brush-forming polymers [138,139] and zwitterionic polymers [140,141,142], as well as the usage of passivating albumin layers.

*Brush-forming polymers.* Coatings of hydrophilic, brush-forming polymers are referred to suppress protein adsorption and reduce interaction between proteins and blood cells on the foreign surface as illustrated in Figure 2A [143,144]. There are two mechanisms that contribute to this effect which are the hydration force and steric repulsion. A tightly bound thin layer of water on surfaces provides a stealth effect due to strong repulsive hydration forces [39], which suppresses protein adsorption. The second component is the steric repulsion force, which is related to compression and entropy decrease of hydrophilic polymeric chains immobilized on surfaces [145]. The repulsive effect is dependent on the density and the molecular weight of the polymer used, as an increasing chain length leads to a decrease of protein adsorption on a functionalized surface [146,147,148]. Covalently bound polymer brushes can be manufactured through two general ways, namely “grafting to”, where polymer chains are immobilized onto the surface or “grafting from”, where the polymerization takes place directly on the surface [149]. Other methods are bulk modification or physical adsorption of block copolymers such as Pluronics [150]. However the disadvantage of “grafting to” is the limited coating density due to steric hindrance during the immobilization process which permits adsorption of smaller proteins among the immobilized macromolecules [151]. Hence, “grafting from” is the more favorable but also more costly variant because of the higher coating density that is achieved.

Polyethylene oxide (PEO), which is (depending on the molecular weight) also known as polyethylene glycol (PEG), is the most investigated compound regarding hydrophilic, brush-forming polymers [152,153]. The simple structural repeat unit of the polyether is able to form a hydration layer, creating a hydration barrier between the surface and the blood, which resists adsorption of plasma proteins to some extent [154]. However, the main effect of PEO surfaces is the steric repulsion force. Here, protein adsorption is also thermodynamically hindered by the compression of surface-bound hydrophilic PEO molecules that decreases their mobility, something which is energetically unfavorable. The length of immobilized macromolecules and their coating density have a high effect on protein adsorption which means that (smaller) proteins may still find adsorption sites, if the surface coating is not dense enough [151,155]. Because of the incomplete surface coverage with higher molecular weight PEG, Ratner et al. developed the concept of tetraglymes through the plasma grafting of small molecular weight PEO to reach a denser coverage, with promising results regarding protein adsorption and blood compatibility [33]. Moreover, PEO coatings can activate the complement system and are not suitable for long-term application due to metal ion-catalyzed oxidation which can lead to their decomposition [156].

*Zwitterionic coatings.* A promising strategy to improve the hemocompatibility of blood-contacting medical devices is modification with zwitterionic molecules (see Figure 2B). These molecules have negatively and positively charged groups but the overall net charge is neutral at physiological pH [58]. Surfaces coated with zwitterions have a high hydration capacity via polar interactions with water molecules leading to excellent protein resistance that is primarily related to repulsive hydration forces [157]. Typical zwitterionic coatings are made of phosphorylcholines, sulfobetaines, and carboxybetaines [48,158,159].

A clinically applied non-thrombotic coating for medical devices, such as cardiovascular devices in dialysis or ventricular assist devices, is the 2-methacryloyloxyethyl phosphorylcholine (MPC) polymer coating [5]. It is a phospholipid-like polymer, mimicking the surface structure of the cellular membrane of red blood and other cells [141,160]. MPC coatings are generated either through physical adsorption or through covalent attachment [161]. It has been reported that surfaces with immobilized MPC show reduced protein adsorption, platelet adhesion, and complement activation [1,160]. The effectiveness of the MPC polymer coating against thrombus formation is based on the creation of a hydration layer through interaction of MPC with water molecules, leading to increased hydrophilicity of the surface and the creation of a strongly repulsive hydration force.

*Passivating albumin layer.* Surface modification with autologous proteins to generate hemocompatible biomaterials may be an elegant method to prevent foreign body responses as illustrated in Figure 2C. Albumin can be suitable for this approach. It is the main protein in blood plasma and, due to its lack of peptide sequences for interaction with coagulation and complement system and cells, is not involved in blood coagulation and immune responses. Furthermore, steric repulsion by bound albumin is suggested for the prevention of protein adsorption and platelet adhesion [162]. As a result, in contrast to fibrinogen, albumin induces less platelet adhesion [58,150]. There are two general ways to fabricate an albumin-coated surface. One is simply based on adsorption on hydrophobic surfaces which can be followed by albumin denaturation and degradation [5]. The other more advantageous method is through albumin-binding compounds, e.g., antibodies, bilirubin, or aliphatic C18 fatty acid chains with subsequent albumin adsorption [32,163,164]. The latter approach has the advantage that albumin is adsorbing in a reversible manner from surrounding plasma maintaining its conformation with low platelet adhesion and complement activation [165].

#### 4.1.3. Textured Surfaces

Modelling of surface topography to create textured surfaces (e.g., forming geometric features, like surfaces with cavities or fibrils) is another possible approach to improve hemocompatibility of blood-contacting biomaterials [166]. Experiments with textured surfaces for the clinical use of medical devices have been performed since the 1960s [167]. The hemocompatibility is due to the way textured surfaces promote the formation of a stable biological lining, called a pseudo neointimal layer of adsorbed and denatured proteins and other entrapped blood components [168]. There are several methods to modify surface topography and to create textured surfaces including the use of sintered titanium or argon plasma etching to prepare micropatterns on titanium oxide layers, solvent casting of PU, using molds of patterned cavities, and particle casting to form geometric features on surfaces [168,169,170]. Medical devices with textured blood-contacting surfaces have generally shown good hemocompatibility [171]. In comparison with non-textured surfaces less thrombus formation was found [172]. However, the formation of a stable neointimal layer on the material is strongly dependent on the topography of the surface. For example, it has been found that the cavity size and distribution of segmented PU has a significant effect on the structure, thickness, and stability of the neointimal layer [168].

### 4.2. Bioactive Surfaces

Biological active surfaces can be an effective method to prevent activation of coagulation or inflammation. There are several strategies for biologically inspired biomaterials, including the incorporation of coagulation inhibitors, as well as antiplatelet and fibrinolytic agents.

#### 4.2.1. Anticoagulant Surfaces

There are different types of anticoagulant agents which are suggested for use as hemocompatible modifications for medical devices. The various anticoagulants, e.g., direct and indirect coagulation inhibitors, anticoagulant proteins, and contact system specific inhibitors have a broad mechanism of action with coagulation factors as the main target.

*Heparin.* The most widespread method for surface modifications with anticoagulants is the immobilization of heparin, an indirect thrombin inhibitor working through binding to ATIII as shown in Figure 2D. The first therapeutic use of heparin took place in the late 1930s for the treatment of deep vein thrombosis. Immobilization of heparin on biomaterials surfaces goes back to the early 1960s as reported by Gott et al. [173] and has been in clinical application for over 30 years [62]. Widely used to increase hemocompatibility of biomaterials, heparin modifications have found application in many medical devices, such as dialysis membranes, vascular stents, or ventricular assist devices [3,174,175]. The anticoagulant activity of heparin is due to indirectly inhibiting thrombin and FXa, as well as other coagulation factors, by catalyzing the inhibitory activity of AT III [60,63,176]. Hence, heparin has the advantage that it works in a catalytic manner and is therefore not consumed. The interaction between heparin and AT III is dependent on a pentasaccharide sequence which is required for the binding of AT III. In addition to its anticoagulant activity, heparin also shows anti-inflammatory properties by inhibiting the complement system and leukocyte activation [81,177].

Many modification strategies have been used to immobilize heparin onto metal or polymer surfaces. These techniques include electrostatic adsorption, based on the interaction between the negatively-charged sulfate groups of heparin with positively-charged groups (e.g., amino groups) on the surface, covalent immobilization in a side-on or end-on manner, integration into hydrogels, or insertion into release systems [62,176,178]. Covalent bonds are obtained through different surface conjugation chemistries, whereby the various functional groups of heparin, like carboxyl groups, are used for the chemical binding reaction [63,179]. The method of immobilization plays an important role with respect to the anticoagulant activity of heparin [180]. Electrostatically bound heparin can be exchanged by other ionic compounds in the blood. Covalently immobilized heparin may lose its anticoagulant activity through structural changes due to chemical conjugation. Another limitation of heparin is its lifetime, which is limited due to its biodegradability in vivo. Moreover, heparin has the tendency to bind various plasma proteins other than AT III (e.g., low density lipoprotein) which may result in a loss of anticoagulant activity [181]. As mentioned above, heparin activity is dependent on a pentasaccharide sequence to activate AT III. However, in commercial heparin preparations only one third of the molecules contains this sequence. To get around this problem, Chan et al. developed a method to increase the pentasaccharide sequence content and consequent anticoagulant activity of heparin coatings by generating a heparin-antithrombin-complex with at least one sequence per heparin [182]. Regarding the surface modification with heparin for medical devices, the Carmeda^®^ BioActive Surface technology should be mentioned. It is one of the most widely applied techniques used commercially. In this method, heparin is only covalently bound to the material surface at its reducing end, therefore leaving the rest of the molecule untethered and preserving its ability to bind antithrombin [62,180].

*Hirudin and hirudin analogues.* Besides heparin, there are various other, less conventional anticoagulants, which have been suggested to be possible candidates to prepare hemocompatible surfaces, which are visualized in Figure 2D. One possibility is the direct thrombin inhibitor hirudin, a naturally occurring peptide which can be isolated from leeches [183]. There are several approaches to hirudin modifications for a variety of substrates, such as polyester, PU, PTFE, nitinol, and polyethylene [184,185,186,187]. For example, Li et al. developed a hirudin coating on polylactide membrane, where hirudin was immobilized through hydrogen bonding interactions showing a good hemocompatibility in vitro [186]. Alibeik et al. described surfaces with anti-thrombotic potential by combining PEG (protein resistance) and hirudin (thrombin neutralization) [188]. However, in contrast to heparin, the interaction between hirudin and thrombin is an irreversible process, which means that each hirudin molecule can only inactivate one thrombin molecule [5].

The synthetic hirudin analogue bivalirudin may be an alternative to hirudin with the advantage of inhibiting thrombin reversibly. Lu et al. immobilized bivalirudin onto stainless steel using a bonding layer of polydopamine [189]. In vitro tests of the hemocompatibility showed prolonged coagulation time and inhibition of the activation of platelets.

*Anticoagulant proteins & contact system specific inhibitors.* Anticoagulant proteins, including thrombomodulin [190,191,192,193], activated protein C [194], or TFPI [195], have been immobilized to biomaterial surfaces of vascular stents or vascular grafts. For example, thrombomodulin has been covalently immobilized to nitinol surfaces which were pre-treated with amino-terminated organosilanes to generate an aminated surface for further reaction [196]. The study showed that the ability to enhance protein C activation remains and only few platelets adhered to the surface. However, degradation in vivo, loss of activity during sterilization, as well as high costs, represented some disadvantages [5]. Furthermore, the bioactivity of covalently immobilized thrombodulin may be reduced due to possible involvement of functional groups for immobilization which are needed for thrombin binding [58].

Other anticoagulants of interest are contact system specific inhibitors which address HMWK, KK, FXIIa or FXIa. For example, corn trypsin inhibitor, an inhibitor of FXIIa, has been applied for immobilization on surfaces [197]. Alibeik et al. [198] coated a PU substrate with a PEG-corn trypsin inhibitor conjugate. The surface showed reduced fibrinogen adsorption and inhibition of FXIIa in vitro. Preliminary in vivo studies with PEG-corn trypsin inhibitor catheters showed good results, but further studies are needed for an eventual application in the medical field [199].

#### 4.2.2. Platelet Inhibitors

Blood platelet inhibitors are another modification strategy to prevent thrombus formation on blood-contacting medical devices. Surface coatings and release systems, targeting adhesion, activation, and aggregation of platelets are based on, e.g., prostaglandin E1, dipyridamole, the immobilization of apyrase, systems eluting GPIIb/IIIa inhibitor abciximab, and nitric oxide (NO)-releasing coatings (see Figure 2F).

Approaches using the lipid prostaglandin E1 to suppress platelet activation and aggregation on biomaterials have been employed for a long time [200,201,202]. The immobilization can take place either in a stable manner or release systems. Chandy and Sharma, to name one example, described a method for the immobilization of prostaglandin E1 on albumin-coated polymer substrates, demonstrating a good antiplatelet activity [202]. Because ADP is among one of the strongest activators of platelets, it is also desirable to prevent this type of activation. Aldenhoff et al. investigated polyurethane surfaces, on which dipyridamole, a synthetic phosphodiesterase inhibitor, which blocks the platelet ADP receptor, was covalently grafted [203]. In vitro studies revealed reduced thrombogenicity related to reduced adhesion of platelets [204,205]. Decreased platelet activity was also shown on polystyrene surfaces with immobilized apyrase, an ADP degrading enzyme [206].

An interesting and emerging approach are NO-releasing or NO-generating materials. NO is a signaling molecule which is released into the blood by the healthy endothelium. The multiple functions of the molecule in vivo, like the prevention of platelet activation and aggregation, as well as anti-inflammation and anti-bacterial behavior, makes it a promising compound regarding hemocompatible biomaterials [181]. There are two main strategies for the local NO production of surfaces. One approach is based on a direct release system, where NO donors like diazeniumdiolates are immobilized [207]. Another option is the application of catalytic agents that use physiological sources for the synthesis of NO. For example, cystein-modified polymers were used for transnitrosation of endogenous S-nitrosogluthatione or S-nitrosoalbumin to achieve release of NO [208,209]. First studies for application of such types of coating have been conducted for coating of cardiovascular stents [210] or small-diameter blood vessels [211].

#### 4.2.3. Fibrinolytic Agents

All of the modifications described above have the goal of preventing thrombus formation, including the activation of blood platelets. An alternative concept may be the generation of clot-lysing surfaces [212]. To realize this strategy two main approaches are possible; mimicking the fibrinolytic mechanism of the vascular endothelium or release systems liberating tPA [181]. It is expected that plasmatic plasminogen, with its lysine-binding sites, can be captured by lysine-functionalized surfaces. Based on this concept, Woodhouse et al. [213] investigated lysine-containing PUs. Here, the lysine was directly immobilized to the surface through sulfonate groups on the PU chains. It has been demonstrated that plasmatic plasminogen was adsorbed in significant amounts and not displaced by other plasma compounds. Another approach, based on lysinized silica surfaces, showed that there is a significant preference for plasminogen adsorption over fibrinogen [214]. Furthermore, Tang et al. reported a copolymer-modified PU surface, based on graft copolymerization of a lysine-containing methacrylic monomer and hydroxyethyl methacrylate (HEMA) which is able to adsorb plasminogen and tPA from blood, activating plasmin generation, and thus, fibrinolysis [215]. These findings make this a suitable approach for potential use in medical devices. Clinically approved plasminogen activators, including streptokinase, urokinase and tPA, are used to treat thrombotic diseases like an ischemic stroke [212]. Thus, concepts based on surface-mediated tPA release systems may be another possibility for fibrinolytic surfaces. Park et al. [216] developed a tPA-loaded porous poly(L-glutamic acid) (PLGA) semi-interpenetrating polymer network hydrogel for regulated tPA release. This hydrogel was generated through free-radical polymerization and cross-linking of PEG-methacrylate through the PLGA network. Results suggest that this system may be a potential delivery system for the local release of active tPA. Despite good approaches, the clinical use of fibrinolytically active surfaces has not yet been realized [5].

### 4.3. Endothelialization

The vascular endothelium (the inner surface of blood vessels) is the only surface that can be truly described as non-thrombotic. Endothelial cells (EC) naturally have antithrombotic properties, such as the presence and release of anticoagulant heparan sulfate, thrombomodulin, and release of NO. Thus, a promising approach to increase hemocompatibility of blood-contacting medical devices would be the recreation or simulation of the endothelial layer to mimic the native anti-thrombotic inner lining of blood vessels (see Figure 2E).

One engineering strategy for the endothelialization of material surfaces is in vitro cell seeding, where autologous or allogenic ECs are harvested and cultured, and subsequently seeded on the blood-contacting device surface before implantation [217,218]. However, in vitro EC seeding is a very time-consuming and cost-intensive procedure, and is challenging because of problems with cell sourcing, cell stability, and cell viability [58]. Moreover, in the case of autologous cells, there is the need of two operative procedures increasing the possibility of contamination and infection. Additionally, the use of allogenic cells can cause rejection [219].

**Table 1:** Examples of commercially applied surface modifications for medical devices.

Modification	Product	Description	Application	Ref.
Diamond-like carbon coating	VentrAssist^TM^, Ventracor		VAD	[1]
	EVAHEART^®^	DLC coating of blood contacting surfaces of the pump	VAD	[220,221]
	Carbofilm^TM^, Sorin Biomedica		Artificial heart valves	[222]
	Diamond Flex^TM^, Phytis	Stainless steel coated with DLC	Stents	[223]
Zwitterionic coatings based on phosphorylcholine	EVAHEART^®^, Sun Medical Technologies	MPC polymer coating of the pump shaft and bearing	VAD	[220,221,224]
	BiodivYsio, Biocompatible	Phosphorylcholine containing copolymer coating	Stents	[224,225]
	TriMaxx^TM^, Abbott	Stainless steel coated with phosphorylcholine	Stents	[226]
	Physio^®^, Sorin Biomedica	Phosphorylcholine-coated tubing	Artificial lung (oxygenator)	[227]
Textured surfaces	HeartMate, Thoratec Corp.	Diaphragm with integral fibrillary texture; textured titanium	VAD	[3,228]
Heparin	DuraHeart^TM^, Terumo Heart	Covalently bonded heparin	VAD	[229,230]
	InCOR^®^, Berlin Heart	CNAS coating ^1^	VAD	[6,62]
	Trillium^®^, Biopassive Surface, Biointeractions Ltd.	Covalently bonded heparin	Cardiopulmonary bypass devices & hemodialysis catheters	[62]
	BIOLINE^®^	Ionically and covalently bonded heparin	Extracorporeal circulation devices & vascular grafts	[62]
	GORE^®^, W. L. Gore and Associates	CNAS coating ^1^	Vascular grafts	[161]
	PROPATEN^®^, W. L. Gore and Associates	CNAS coating ^1^	Vascular grafts	[161]
Endothelialization	Genous^TM^, OrbusNeich Medical Technologies	Covalently bound anti-CD34 antibody layer	Stents	[219]

^1^ Carmeda Bioactive Surface (CNAS) Technologies; heparin is covalently bound by endpoint attachment.

A promising method for setting these limitations may be in vivo self-endothelialization, induced by endothelial progenitor cells [217,219]. The technique is based on the immobilization of endothelium specific antibodies, DNA or peptide aptamers, or anti-cadherin on the materials surface which can capture endothelial progenitor cells from the circulating blood of patients [231]. This allows their attachment and subsequent differentiation, leading to the growth of an EC layer on the blood-contacting surface. However, this method is not very selective, so that in addition to ECs, other cells can also grow on the surface [232]. To avoid restenosis (growth of vascular smooth muscle cells) on the biomaterial, modifications with anti-proliferative drugs have been suggested for the devices [233].

## 5. Conclusions

The studies on fundamentals and development of blood compatible surfaces have a long history. Fundamental studies on protein adsorption, blood coagulation and response of innate and adaptive immunity have provided a deeper understanding on how blood components interact with man-made materials which also helped to design better hemocompatible surfaces. Protein adsorption has been considered a key element depending on surface properties of biomaterials leading to the design of either protein resistant or selective surfaces. On the other hand, several concepts of bioactive surfaces have been developed seeking primarily to mimic the anti-coagulant functions of endothelial cells by immobilization of heparin or release of NO which represents some of the anticoagulant function of these cells or covering surfaces directly with them to generate the ideal blood compatible lining of the device. The choice of method will depend on several aspects, such as duration of application related to the stability and functionality of coatings, the flow conditions, as well as the costs incurred for the device.

## Data Availability

The study did not report any data.
